# Analytical Modelling of Temperature Distribution in SLM Process with Consideration of Scan Strategy Difference between Layers

**DOI:** 10.3390/ma14081869

**Published:** 2021-04-09

**Authors:** Linger Cai, Steven Y. Liang

**Affiliations:** Georgia Institute of Technology, Woodruff School of Mechanical Engineering, Atlanta, GA 30309, USA; steven.liang@me.gatech.edu

**Keywords:** SLM, analytical modelling, temperature distribution

## Abstract

In the practical selective laser melting (SLM) manufacturing process, the scan strategy often varies between layers to avoid overlapping of the melted area, which affects the residual stress and deflection of the final build. Yet not much modelling work has been done to accommodate the angle between layers. The paper proposed an analytical thermal model to address the scan strategy difference, such as laser scan direction difference between layers, which brings the model closer to the practical scan situation. The analytical transient moving point heat solution is adopted in this model. The laser movement is first considered in a laser coordinates, which originates at the laser radiation spot, and then transferred into a stationary coordinate, which originates at the starting point of the build. The model takes account of multi-track and multi-layer effect by considering thermal property changes caused by remaining heat, which is further adopted for temperature distribution calculation. The scan direction difference leads to different laser path at each layer, and alters heating and cooling time for a specific point on the build. The proposed model is validated by comparing the predicted melt pool geometries to documented experimental data. The effect of scan direction difference between layers is further discussed in the later part. It is found that the uni- and bi- directional scan leads to diverse temperature profile but its effect on melt depth is not significant. Although the laser rotation angle between layers leads to changes in the melt depth, it is not in a large scale. The proposed model shows that scan strategy does not change melt pool geometry in a significant scale but affects the thermal profile as well as thermal history. It can be used as a step for further modelling work for porosity and deflection.

## 1. Introduction

Additive manufacturing (AM) gained lots of attention in the manufacturing industry due to its ability to produce complex geometries and lighter weight parts, which makes it attractive to aerospace, automobile, and bio-medical industries [1,2]. With appropriate production strategy, it is also widely integrated with traditional manufacturing [3]. AM offers shorter lead time and less production costs when low-production volume is considered [4]. In some cases, it is also an alternative for conventional production of injection molding and core fabrication in foundry industry [5,6]. Selective laser melting (SLM) is a sophisticated powder-bed AM technology where a scanning laser is used to sequentially melt layers of powdered metal [7]. This process involves in rapid heating and solidification. The high temperature gradient produced in the process leads to complicated physiochemical and thermodynamic phenomena within a melt pool, such as laser absorption, heat transfer and scattering [8]. The lack of control of the temperature results in undesired cracking [9], residual stresses [10], and part distortion [11]. Therefore, the understanding and prediction of the temperature during the SLM process is of great importance.

The numerical modelling based on finite element method (FEM) is widely used. Li et al. [12] used FEM to model the temperature profile of SLM fabricated 316L steal. They started from the single-track heat transfer model and further investigated the temperature and the residual stress filed in the multi-track multi-layer process. They included the temperature dependent material properties as well as the vaporization and shrinkage processes. Liu et al. [13] established a 3D finite element model for temperature prediction of multi-laser selective laser melted AlSi10Mg part. In their study, the laser spot moved step by step with specific exposure time and point distance. They found that the laser re-melting significantly changed the characteristics of the melt pool. As the re-melting time for a specific point was increasing, the melt pool was enlarged. Khan et al. [14] built their FEM model focusing on different support structures during SLM building. They studied the heat dissipation in AlSi10Mg parts built with solid base, supporting structures, and loosely placed powder. Their results showed that the melt pool depth increased with layer addition. FEM is used in many researches, yet the complexity of the AM process requires a significant amount of computational time via FEM.

Analytical models, in contrast, have the advantage of computational efficiency without compromising its prediction accuracy and applicability [15,16]. The instantaneous moving source was proposed by Jeager [17]. Rosenthal [18] developed the quasi-steady-state solution for a point heat source, where a multiplier function was introduced. Based on that, Eager and Tsai [19] further considered a travelling Gaussian distributed heat source and derived a solution for the weld shape. van Elsen et al. [20] derived the analytical uniform heat source solution, the moving heat source solution, as well as the semi-ellipsoidal moving heat source solution based on Carslaw and Jaeger [21]. In the model, a semi-infinite media was assumed. The material was assumed to be isotropic and homogeneous. The solutions derived were in steady state and the part dimension was neglected. One deficit of the analytical solution at this stage was its neglection of the temperature dependent material properties, which played an important role in the practical case. To consider the temperature dependent material property, the solution to the heat governing equation would be non-linear and hard to develop a closed form solution.

To accommodate the problem mentioned above, a semi-analytical solution has been proposed, where the material properties was assumed to change with temperature and iterated with calculated temperature field. Steuben et al. [22] called it enriched analytical solutions and proposed that the material properties at one calculation step was affect by the temperature calculated at last step. Their method achieved sufficient accuracy and shows reasonable result in comparison with FEM calculation. This method was also adopted by other researches. Mirkoohi et al. [23] investigated the accuracy and applicability of five different heat source models. In their analysis, the temperature dependent material properties, such as thermal conductivity, specific heat, and density, were included. These parameters were determined by the temperature calculated at last laser step. Mirkoohi also predicted the balling effect by comparing the melt pool length to depth ration to π. The prediction result was compared with experiments done with Ti-6Al-4V.

Ning et al. [24] introduced an in-process temperature model on a stationary coordinate and included laser power absorption rate, latent heat of fusion, scan strategy of uni- or bidirectional, as well as the power packing porosity. Experiments done with Inconel 625 were used for validation. The introduction of the stationary coordinates provided a possibility to include the part dimension into the temperature calculation, which promoted the applicability of the analytical model. Ning et al. [25,26] further adopted the heat sink solution to accommodate the heat loss at the part boundary. The final solution was considered as a superposition of a moving point heat source solution and multiple heat sink solutions. This methodology was used for both powder bed metal setting and powder feed setting. Experiments done with Inconel 718 were used for validation.

**Figure 1:**
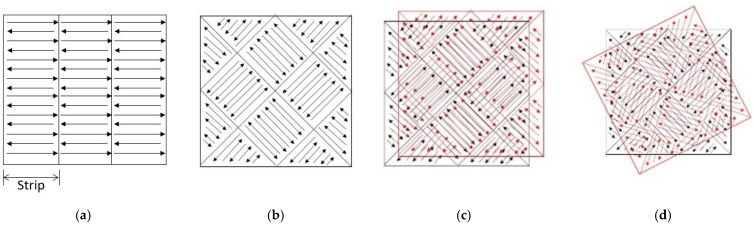
Illustration of the (**a**) “strip scan strategy”; (**b**) “island scan strategy” at single layer; (**c**) displacement between layers; (**d**) rotation between layer. The black config represents the scan strategy of nth layer and the red config represents the scan strategy of (n + 1)th layer.

Unlike model simplifications, the industrial production process often involved complicated scan strategies, especially for parts with complex geometries. Within a layer, the strip strategy was wildly used. The printed region is divided into several strips and laser came back and forth within that strip as shown in Figure 1a. An “island scanning strategy” was also wildly used for residual stress balancing during the build [27]. The building layer in SLM was divided into small squares, in which the laser scans uni-or-bidirectional. Between layers, a displacement [27] or a rotation [28], as shown in Figure 1, was often applied to reduce the residual thermal stress by avoiding repeated heating and solidification at the same location on the part. Wang et al. [29] investigated three self-developed scan strategies with 316L steel. It was found that the scan pattern that mostly overlapped with previous layer produces a large amount of residual stress in the overlap area. The scan pattern which was rotated between layers improved the distribution of residual stress and therefore reduced the final deformation of the build part. They also pointed out that the scan length and the direction played an important role in deformation control. Ali et al. [30] studied the effect of scan strategy on residual stress and mechanical properties of the SLM build Ti-6Al-4V parts. They showed that with the same build, a long bidirectional scan strategy on one layer with 90-degree scan direction between layers led to least amount of residual stress. Guo et al. [31] investigated the effect of scan strategy on surface quality, microstructure, and mechanical properties of the SLM build pure tungsten parts. They found that scan strategy significantly changed the surface morphology as well as the compressive stress. Rashid et al. [32] investigated on density and metallurgical properties of 17-4PH parts with different scan strategy. Their result showed that double scan strategy led to better part density and higher hardness than single scan strategy did. They also found that proper heat treatments of the as-built sample provided better hardness as well.

Although scan strategy is of importance in the final part properties, the modelling of scan strategy is less studied. Perry et al. [33] proposed a thermo-mechanical model to predict the residual stress in SLM. They used the volumetric Gaussian heat source model to address the temperature calculation. They used a constant point overlap factor to make sure that the heat flux transverses across the whole scan path without gaps. Song et al. [34] applied FEM to study the effect of scan strategy on the temperature profile and the residual stress of SLM build Ti-6Al-4V part. They used moving Gaussian heat source for the temperature calculation. In different simulation, they assumed different angles between the laser scan directions of the two layers. It was found that in the simulation, the temperature profile was significantly changed across the part. However, the melt pool geometries did not show significant difference with respect to the changing angles.

For analytical modelling, Ning et al. [24] included calculation with uni- or bi-directional scan on one layer. However, there was not a model built to address the scan direction rotation between layers in SLM. Considering the importance of the rotation angle to the various aspects [28,29,30,31,32] of the build, it is important to include it into the analytical model. This paper proposes an analytical thermal model for SLM that considers the scan direction rotation between layers. The analytical transient moving point heat solution is adopted in this model. A stationary coordinate is also introduced. Temperature dependent material properties are considered in the model. The scan direction rotation is considered as the thermal property difference introduced by the temperature profile from previous layer. Here in the model, the porosity of the material during melting is not considered. The predicted temperature profile is validated by comparing predicted melt pool geometry to experimental data published in literatures. An investigation of the effect of the rotation angle is also performed.

## 2. Methods

In this model, a semi-infinite media is assumed, and the material is assumed to be isotropic and homogeneous. The laser spot is considered as a moving point heat source. The temperature solution induced by a moving heat source can be derived from the heat balance governing equation as following:

(1) $$\frac{\partial \rho u}{\partial t}\;+\;\frac{\partial \mathrm{Ev}}{\partial s}\;=\;\nabla \cdot \left(k\;\nabla T\right)\;+\;\dot{Q}$$

where ρ is the material density, u is the internal energy, E is the enthalpy, v is the heat source velocity, k is the thermal conductivity, and Q is the volumetric heat source. t represents time and s represents the distance between the heat source to the point of interest. T is the temperature.

Take V= 0, Equation (1) becomes

(2) $$\frac{\partial \rho u}{\partial t}\;=\;\nabla \;\cdot \;\left(k\;\nabla T\right)\;+\;\dot{Q}$$

Because of the first law of thermal dynamic $du=C_{p}dT$, the above equation becomes

(3) $$\frac{\mathrm{dT}}{\mathrm{dt}}\;=\;\frac{k}{C_{p}\rho }\;\left(\frac{\partial^{2}T}{\partial x}\;+\;\frac{\partial^{2}T}{\partial y}\;+\;\frac{\partial^{2}T}{\partial z}\right)\;+\;\dot{Q}$$

where $C_{p}$ is the specific heat of the material. Now, set the thermal diffusivity $\kappa =\frac{C_{p}\rho }{k}$, the following equation is obtained.

(4) $$\frac{\mathrm{dT}}{\mathrm{dt}}\;=\;\frac{1}{\kappa }\;\left(\frac{\partial^{2}T}{\partial x}\;+\;\frac{\partial^{2}T}{\partial y}\;+\frac{\;\partial^{2}T}{\partial z}\right)\;+\;\dot{Q}$$

The solution of the point heat source has been developed by Carslaw and Jaeger [21], which is expressed as

(5) $$\;\Delta T\;\left(x,y,z,t\right)=\;\frac{Q}{8{\left(\pi \kappa t\right)}^{\frac{3}{2}}}\;e^{-\left[{\left(x-\;x\prime \right)}^{2}+{\left(y-\;y\prime \right)}^{2}+{\left(z-\;z\prime \right)}^{2}\right]/4\kappa t}$$

This expression gives the temperature change of point (x,y,z) at time t due to a point heat source applied at ( x′, y′, z′) at time  t′=0.

Now consider a laser with power P and scanning velocity V generates a heat input at previous time t′. The total heat amount in flow is Pdt η, where η is the laser absorption rate. Then the strength of heat is $\frac{P\eta \mathrm{dt}}{{\rho c}_{p}}$. Here we assume that the heat source moves along x direction, so  y′=0, z′=0. For a time interval dt′, the temperature change induced is given as

(6) $$\Delta T\left(x,y,z,t\right)=\;\frac{P\eta \mathrm{dt}\prime }{8{\rho c}_{p}{\left[\pi \kappa \left(t-\;t\prime \right)\right]}^{\frac{3}{2}}}\;e^{-\left[{\left(x-V\left(t-\;t\prime \right)\right)}^{2}+y^{2}+z^{2}\right]/4\kappa \left(t-\;t\prime \right)}$$

To get the temperature at curtain point (x,y,z) at time t due to heat input of laser at time t′, integrate above equation from 0 to t regarding t′, and the solution is the following

(7) $$\Delta T\left(x,y,z,t\right)\;=\;\frac{P\eta }{8{\rho c}_{p}{\left[\pi \kappa \right]}^{\frac{3}{2}}}\;\overset{t}{\underset{0}{\int }}e^{-\left[{\left(x-V\left(t-\;t\prime \right)\right)}^{2}+y^{2}+z^{2}\right]/4\kappa \left(t-\;t\prime \right)}\mathrm{dt}\prime$$

The solution can also be expressed as

(8) $$\Delta T\left(x,y,z,t\right)\;=\;\frac{P\eta }{2{\mathrm{Rk}\pi }^{\frac{3}{2}}}e^{\frac{\mathrm{Vx}}{2\kappa }}\;\overset{\infty }{\underset{\frac{R}{2\sqrt{\kappa t}}}{\int }}e^{\left[-\xi^{2}-\left(\frac{V^{2}R^{2}}{16\kappa^{2}\xi^{2}}\right)\right]}d\xi$$

where $R=\sqrt{x^{2}+y^{2}+z^{2}}$, and ξ is an integration variable.

Latent heat of fusion is taken into consideration because of its significant effect on the melt pool dimension [21]. The temperature drop caused by phase transformation is obtained with heat integration method and is expressed as [35]

(9) $$\Delta T\;\left(x,y,z,t\right)\;=\;\frac{L_{f}}{C_{p}}$$

where $L_{f}$ is the latent heat of fusion.

**Figure 2:**
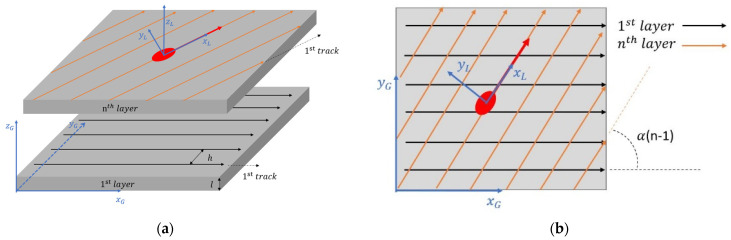
Illustration of the build with laser scan direction rotation between layers. (**a**) general view; (**b**) top view. h is the hatching space between layers. α is the laser scan direction rotation angle between each layer. l is the layer thickness.

The above analysis happens in a moving coordinate, in which the laser radiation spot is the origin, shown as coordinate $X_{L}Y_{L}Z_{L}$ as in Figure 2. A general coordinate $X_{G}Y_{G}Z_{G}$, whose origin locates at the starting point of the build, is also introduced label the point on the build. The layer scan direction rotates α degrees between each layer. For the $n^{\mathrm{th}}$ layer, the total rotational angle between the current layer and the first layer is expressed as

(10) φ = α(n−1),

To express a point $\left(x_{g},y_{g},z_{g}\right)$ on the build in the laser coordinate, a matrix transformation is applied. $\left(x_{l},y_{l},z_{l}\right)$ is the point.

(11) $$\begin{array}{c} x_{l}\\ y_{l}\\ z_{l} \end{array}=\;\left[\begin{array}{ccc} \cos\left(\varphi \right) & \sin\left(\varphi \right) & 0\\ -\sin\left(\varphi \right) & \cos\left(\varphi \right) & 0\\ 0 & 0 & 1 \end{array}\right]\begin{array}{c} x_{g}\\ y_{g}\\ z_{g} \end{array},$$

The temperature rise of the point of interest is then calculated based on Equations (7) and (9).

In calculation, the initial temperature of the powder is assumed to be room temperature. At this moment, for a point $\left(x_{p},y_{p},z_{p}\right)$, its temperature at time $t_{0}$ is $T_{\mathrm{room}}$. Its material properties can be expresses as

(12) $$k_{0}\;\left(x_{p},y_{p},z_{p},t_{o}\right)\;=\;k\left(T_{\mathrm{room}}\right)\;{\mathrm{Cp}}_{0}\;\left(x_{p},y_{p},z_{p},t_{o}\right)\;=\;\mathrm{Cp}\left(T_{\mathrm{room}}\right)$$

As the laser starts to scan to the step, the material is melted because of the energy it deposited, which created a heat affected zone (HAZ) and a melt pool. Equation (8) is then used to calculate a temperature profile, expressed as $T^{0}.\;$As the laser goes to the next step, the HAZ created by previous step does not have enough time to drop back to room temperature. The remaining heat leads to thermal property difference at that area, which affects the temperature calculation for the next laser step.

(13) $$k_{1\;}\left(x_{p},y_{p},z_{p},t_{o}+\Delta t\right)\;=\;k\left(T^{0}\right)\;{\mathrm{Cp}}_{1}\;\left(x_{p},y_{p},z_{p},t_{o}+\Delta t\right)\;=\;\mathrm{Cp}\left(T^{0}\right)$$

Here Δt represents the time difference between two assumed laser steps, which is assumed to be 2.5 ms in this calculation. For $m^{\mathrm{th}}$ laser step, the material properties are expressed as

(14) $$k_{m\;}\left(x_{p},y_{p},z_{p},t_{o}\;+\;m\;*\;\Delta t\right)\;=\;k\left(T^{m-1}\right)\;{\mathrm{Cp}}_{m}\;\left(x_{p},y_{p},z_{p},t_{o}\;+\;m\;*\;\Delta t\right)\;=\;\mathrm{Cp}\left(T^{m-1}\right)$$

This method has been adopted by Steuben et al. [22] and Mrikoohi [23] to avoid the nonhomogeneous solution when solving the heat equation. In both work, it has shown sufficient accuracy comparing to both FEM results and experimental results.

**Figure 3:**
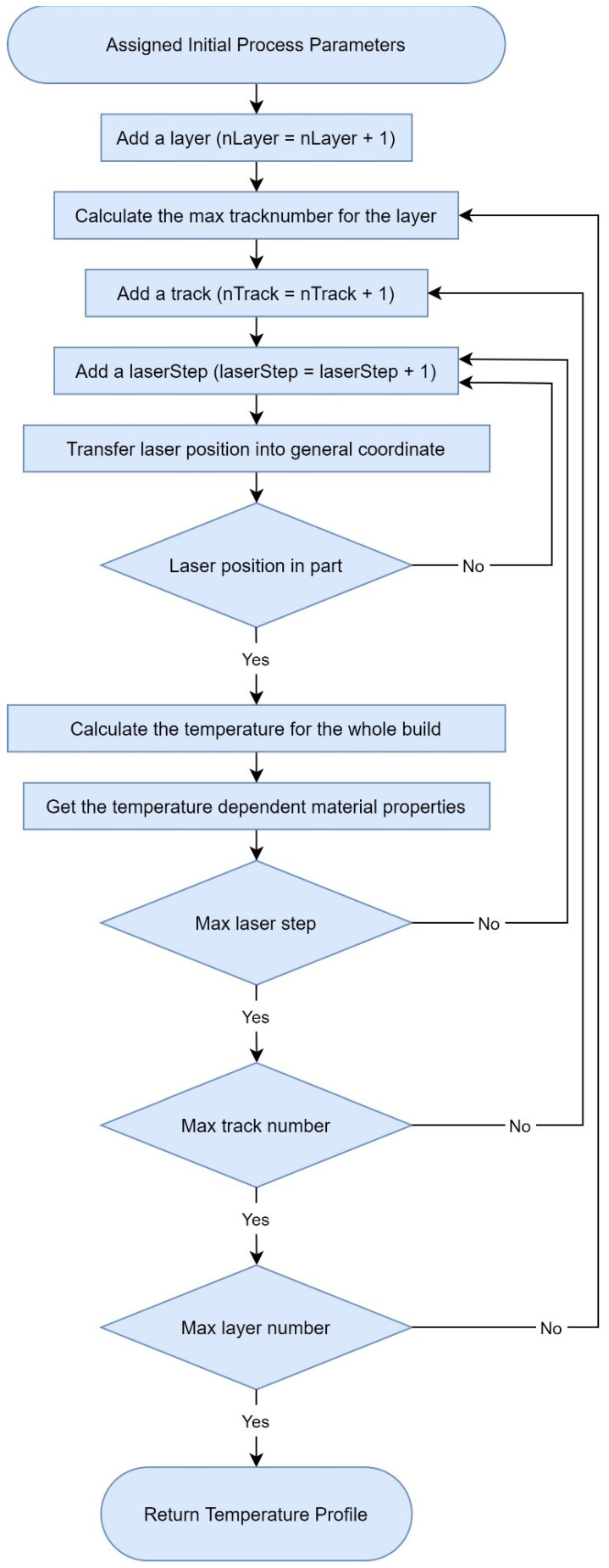
Flow chart for the calculation process.

Here in this model, the laser spot is assumed to be a dimensionless point, which means that the diameter of the laser spot is not considered. The scan direction of the first layer is assumed to be parallel to $X_{G}$ axis. Track numbers on one layer is calculated based on the rotation angle and the hatching space. The flow chart of the calculation process is illustrated in Figure 3. Both multi-track and multi-layer effects are considered. A continuous laser scan without gap time between tracks and layers is assumed in the proposed model.

## 3. Results

The proposed model is validated by comparing the predicted melt pool geometry to the experimental measured ones with AlSi10Mg. A MATLAB program is used to implement the calculation. The material properties of AlSi10Mg are shown in Table 1. The thermal conductivity and specific heat are considered as temperature dependent. Because the melting process involves in material phase changes, the latent heat of fusion and thermal properties beyond the melting temperature are also considered.

Rosenthal et al. [39,40] produced a series of AlSi10Mg specimens with EOSINT M280 system. The powder used is pre-alloyed AlSi10Mg powder with particle size in the range of 15–30 μm. The applied laser power is 400 W and scan speed is 1000 mm/s. The hatching space is 200 μm. The layer thickness is 60 μm. A strip scan strategy, as shown in Figure 1a, with 8 mm strip and 67-degree rotation between layers is used. The macro structure pictures of the SLM build AlSi10Mg published is processed with ImageJ to get the average melt pool depth, which is 70.52 μm. The average melt pool width is measured in the same way and the resultant average melt pool width as 131.66 μm.

**Figure 4:**
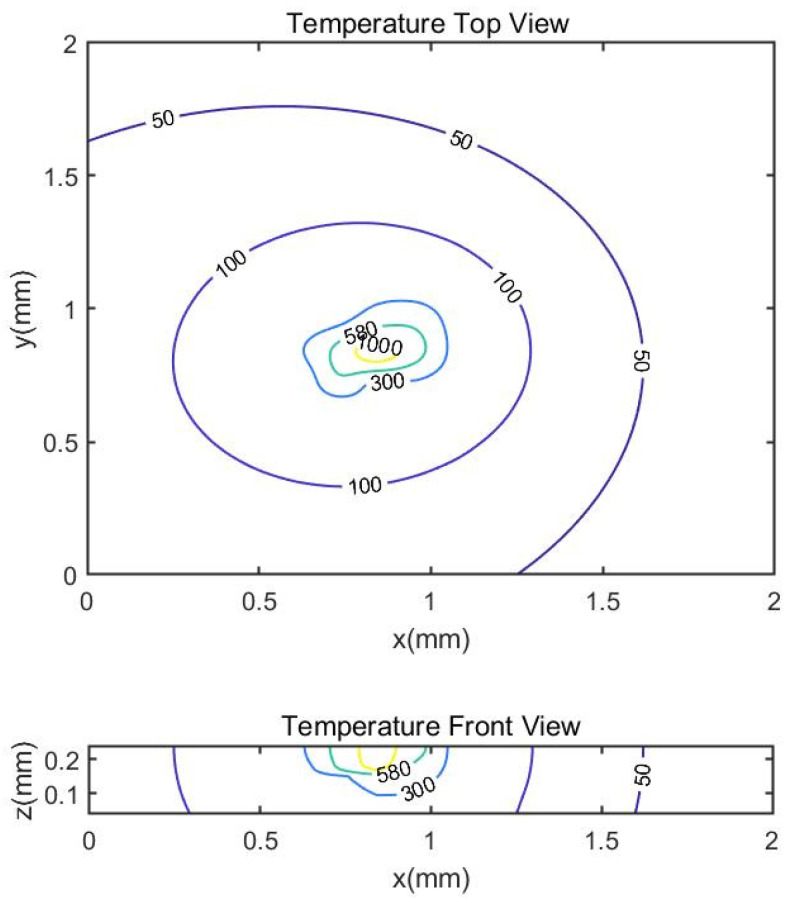
Predicted temperature profile in top and front view with laser power 400 W, scan speed 1000 mm/s, hatching space 200 μm and layer thickness 60 μm. The rotation angle between two successive layers is 67 degrees.

**Figure 5:**
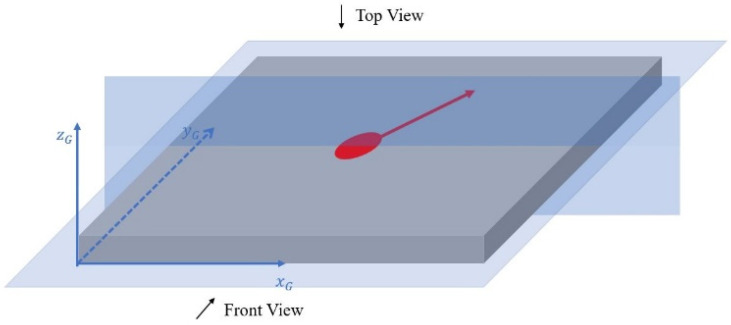
Illustration of the top and front view of prediction.

Based on the model proposed in Section 2 with rotational angle set to be 67 degrees, Figure 4 shows the predicted temperature profile in both top view and side view, which is based on general coordinate as shown in Figure 5. Without specific mention, the figures shown in this work are in general coordinate for the following content. With the same process parameters and the absorption rate set to 0.24, the predicted melt pool depth is 66.33 μm and the predicted melt pool width is 132.66 μm. As there is no additional data set given in this experiment, one indicator for the validity of the model is to compare the ratio of the melt pool depth to the melt pool width. It is seen that the predicted ratio is 0.50, which is close to the ratio in from the experiment, 0.53. The predicted melt pool depth and width are in good agreement with the experiment measured ones.

**Figure 6:**
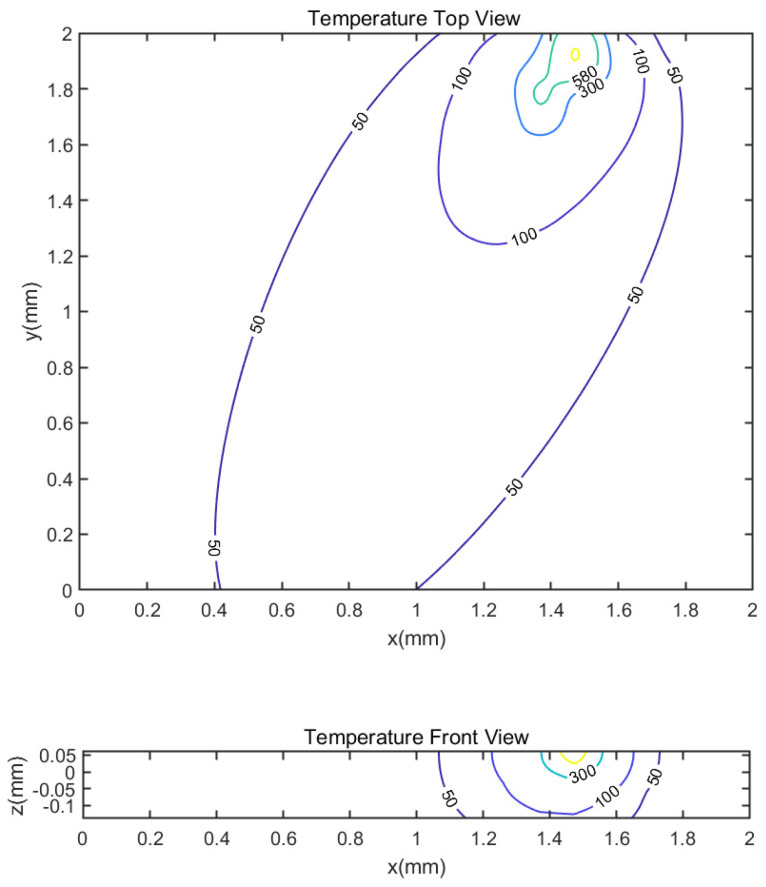
Predicted temperature profile in top and front view with laser power 195 W, scan speed 700 mm/s, hatching space 0.17 mm and layer thickness 30 μm. The rotation angle between two successive layers is 67 degrees.

To further validate the proposed model, similar comparison is done to the another set of experimental data published by Krishnan et al. [41,42]. They use the EOS M270 Xtended machine to produces a set of specimens with AlSi10Mg powder. On the same layer, a bidirectional scan strategy is applied. Between layers, the scan direction rotates 67 degrees with respect to the direction in the previous layer. Several specimens are polished and etched with Weck’s reagent for image analysis, which shows the melt pool geometries. The layer thickness for the experiments is 30 μm for all the specimens. Other process parameters are shown in Table 2, as well as the measured melt pool depth. The predicted temperature profile with process parameters of Sample 3 is shown in Figure 6.

The laser absorption rate varies in different experimental environment and it is difficult to measure directly in the experiments [43]. In SLM, the metal powder layer is thin, and energy is typically absorbed in the upper layer nonuniformly. The absorption rate is significantly affected by the size distribution of the powder and their geometries, which inversely affected the melt dynamic [44]. As no further information is given about the powder used in the mentioned literature, the laser absorption rate is viewed as a variable that needs to be calibrated. The experimental data from Sample 4 is used to calibrate the absorption rate, which is determined as 55%. With the specific absorption rate determined, the predicted melt pool depth is also shown in Table 2 to be compared with the experimental data. The predicted depth is 62.2 μm with lower laser scanning speed, which is deeper than the one with higher laser scanning speed.

Calculated melt pool depth assuming 0 degree angle between layers are also presented in Table 2. Comparing to 67 degree case, the model suggests deeper melt pool depth in 0 degree case. It also shows the trend that with other parameters to be the same, the higher the scan speed, the shallower the melt pool. A further discussion to the effect of laser parameters as well as the rotational angle between layers is placed in the following section.

## 4. Discussion

### 4.1. The Effect of Laser Power and Scan Speed to the Melt Pool Depth

**Figure 7:**
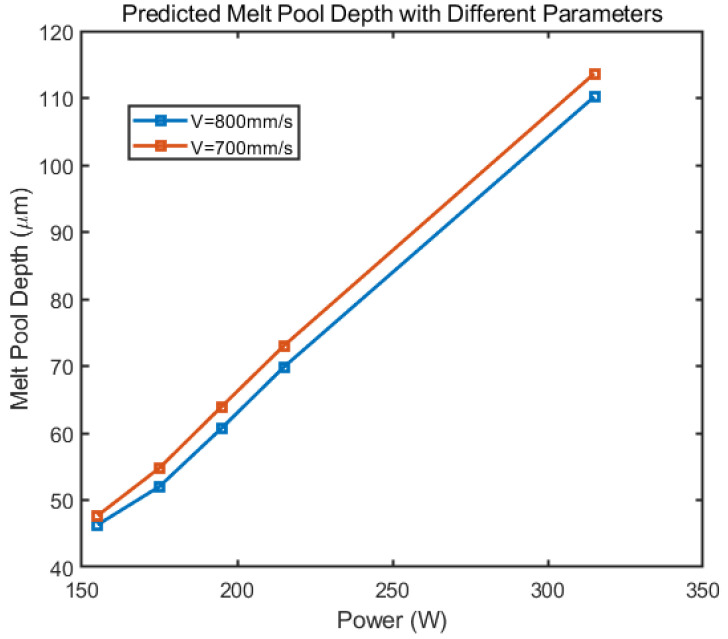
Predicted melt pool depth with different assumed power. The layer thickness 30 μm, and hatching space 0.17 mm.

Figure 7 shows the predicted melt pool depth with different power with rotational angle to be 67 degrees between layers. The assumed layer thickness is 30 μm and the hatching space is 0.17 mm. The model predicts a linear relationship between the applied power to the predicted melt pool depth under given process parameters. The increase in the laser power provides more heat dispensed into the material and thus deepens the melt pool. With increasing scan speeds, the melt pool depth decreases accordingly. This happens because the increasing scan speed results in less time for the material to absorb the heat, which results in less melt pool depth. It is intuitive that both more intensive laser power and slower scan speed increase the melt pool depth, which have also been investigated by other researchers in multiple situations [24,33,34]. However, the current model does not account for possible porosity or balling effect due to incomplete melt nor the keyhole effect due to vaporization of the inner material [45]. Mirkoohi [23] set this criteria as the ratio for the melt pool length and depth to be π for balling effect to occur. Yet there is not a strong reasoning for that. In reality, the melt pool length is usually hard to measure as it will merge into a long track path. The judgement criteria for porosity and keyhole formation in analytical model has not been fully developed.

### 4.2. The Effect of Uni- or Bi-Directional Scan Direction on the Same Layer

**Figure 8:**
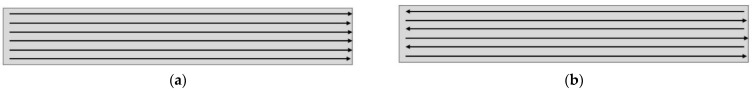
Illustration of the scan strategy at the same layer (**a**) unidirectional scan; (**b**) bidirectional scan.

**Figure 9:**
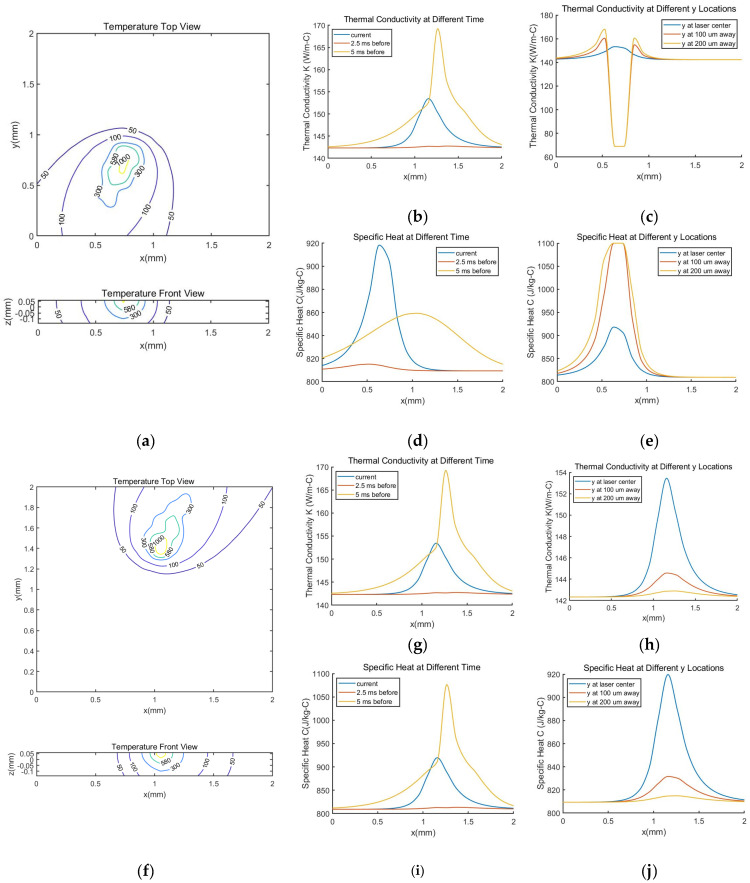
Simulated temperature profiles and corresponding thermal conductivity and specific heat results at 46.3 ms after the beginning of the scan. The applied laser power is 195 W, scan speed 700 mm/s, hatching space 0.17 mm and layer thickness 30 μm. The rotation angle between two successive layers is 67 degrees. (**a**–**e**) are results for the unidirectional scan and (**f**–**j**) are results for the bidirectional scan.

For the scan direction on the same layer, there are usually two options: unidirectional scan and bidirectional scan as shown in Figure 8. Figure 9 shows simulated temperature profiles at 46.3 ms after the beginning of the scanning with uni-directional and bi-directional scan strategies. The same process parameters as Sample 3 in Table 2 are applied to the simulation. For both cases, the laser is on its seventh track at the second level at this instant. The predicted melt pool depths are close as well. For unidirectional scan, the melt depth is 62.4 μm and for bidirectional scan, the melt depth is predicted as 62.2 μm. However, temperature profiles across the 4 ${\mathrm{mm}}^{2}$ pad are significantly different as shown in Figure 9a,f. This leads to underlying thermal property differences, which are also shown in Figure 9. In Figure 9c,e, there is a significant drop of the value of the thermal conductivity and the specific heat. This is due to the melting of the material. As AlSi10Mg melted at that location, phase change occurs, and the thermal properties applied need to be the one for liquid status, where the value of specific heat and thermal conductivity appear to be flat according to the material model adopted as in Table 1.

The effect of thermal property change is not significant when the laser spot is at the inner area of the part. However, these changes have impact at the turning points of the laser scan. For the unidirectional scan strategy, as one track is finished, depending on the track length, the temperature of the starting point for the next track may go back to near the room temperature. However, for the bidirectional scan strategy, the effect of the previous track is more significant. For the bidirectional scan, the material at the starting point of ${\left(n\;+\;1\right)}^{\mathrm{th}}$ track may still be liquid when laser starts passing the ${\left(n\;+\;1\right)}^{\mathrm{th}}$ track. As the thermal properties, such as thermal conductivity and specific heat experience, experience a value drop near the melting temperature, the heat does not dispense to the surrounding material as well as in solid surrounding. This will deepen the melt pool at that point. This is consistent with the simulation results from finite element model [46,47], which indicated that the bidirectional scan strategy led to deeper melt pool depth. The difference in melt pool geometry affects the microstructure of the build and further has influence on the mechanical properties of the build. In the experiment, without the outer contour, the bidirectional scan leads to higher yield strength comparing to the unidirectional case [48]. The relationship between the melt pool geometry to the microstructure, such as grain size and cell size, is not in the scope of current paper, yet this model could serve as a base for the microstructure prediction.

### 4.3. The Effect of Rotation angle on the Temperature History

**Figure 10:**
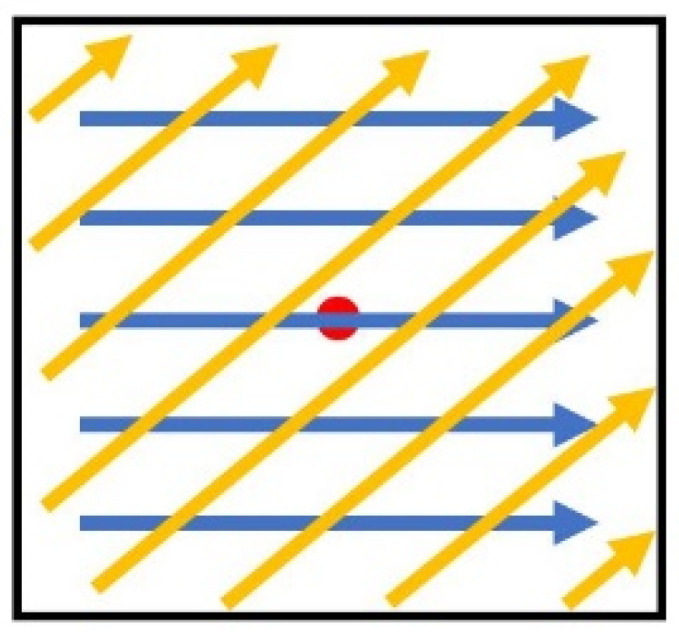
Illustration of how rotational angle changes the laser track on one layer. A blue line represents laser path with 0 degree rotation and a yellow line represents laser path with a rotation angle. The red dot represents the point of interest.

**Figure 11:**
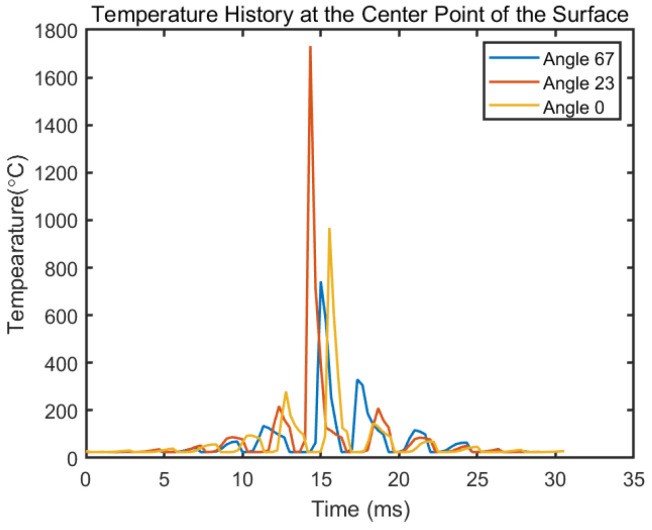
Temperature history of the center point on the fifth layer on a 4 mm^2^ square island with and without different rotational angle between layers. The process parameters are laser power 195W, laser scan velocity 800 mm/s, layer thickness 30 μm, and hatching space 0.17 mm.

The rotational angle between layers changes the total number of tracks on single layer, as well as the scan length of one track, as shown in Figure 10. The laser path illustration is similar to the presentation in Figure 2b. With a 2 mm by 2 mm square island assumed and same process parameters used in Sample 4 in Table 2, a temperature history of the center point at the surface with different rotational angle is given in Figure 11. It shows that with different rotation angle between layers, the point of interest goes through a significantly different temperature history. This will later affect the residual stress and deflection, but these effects are not in the scope of current paper. The different peaks on a single line reflect to the time where the laser spot is closest to the of the point of interest. With a different rotational angle, the center point experiences a different heating and cooling cycle. Because of the existence of the hatching, the layer radiation spot may not go exactly through the center point, as shown by yellow line in Figure 10. The different distance between the laser radiation spot to the point of interest leads to the different maximum temperature in the entire temperature history.

**Figure 12:**
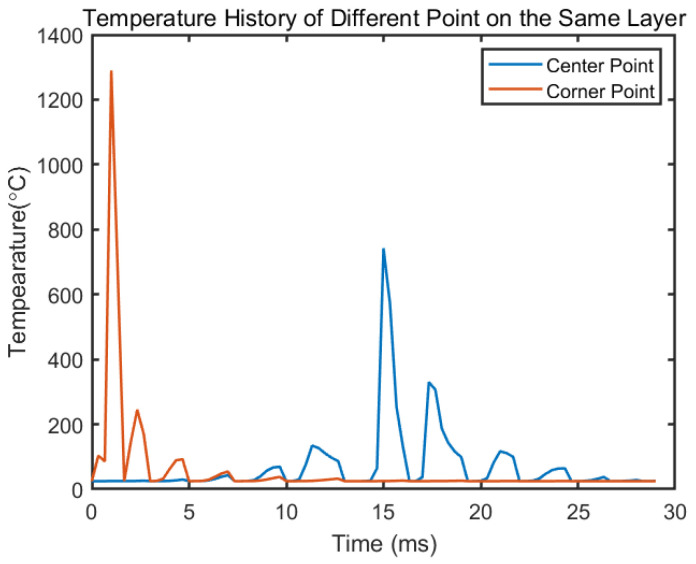
Temperature history of different point on the fifth layer on a 4 mm^2^ square island with the same process parameter.

**Figure 13:**
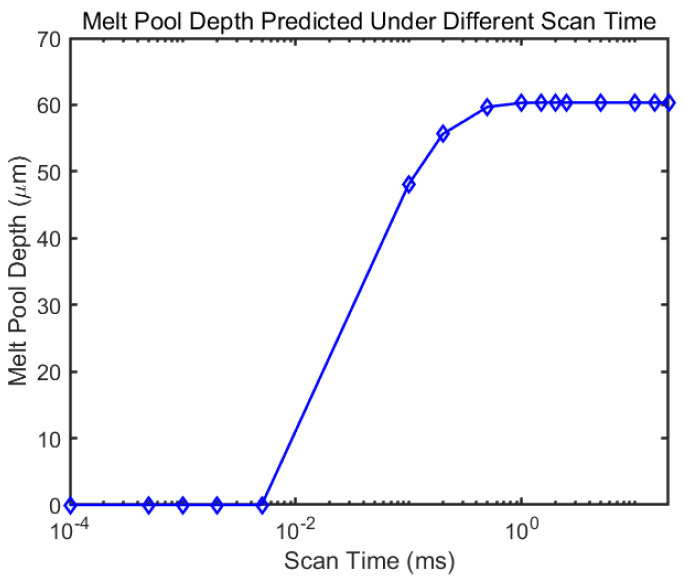
The predicted melt pool depth evolution with continuous scan time.

The temperature history of the center point and the corner point with same process parameters are given in Figure 12. This corner point is close to when the laser begins to scan the third layer, so the peak temperature happens at the beginning. However, as the laser goes far away, the temperature of that specific point goes back to room temperature. Because of the rotational angle, the length of first several paths at the layer do not have the same length, which leads to different time to reach a peak. For the corner point, the first peak reaches at 0.33 ms. The second peak is reached at 1 ms. The third peak comes at 4.7 ms and the fourth comes at 7 ms. These short scan path may not leave enough time for the laser to fully melt the material and form a stable melt pool. With a 25 ${\mathrm{mm}}^{2}$ island size, the corresponding temperature peak comes at 1.88 ms, 3.75 ms, 6.25 ms and 9.37 ms. The large island size elongates the track length, which requires longer time to finish a track. There are researches showed that shorter scan length may reduce the residual stress [49], yet it is not always beneficial. As shown in Figure 13, it takes about 1 ms to form a stable melt pool under given process parameters. A track takes less time than that may lead to incomplete melt. Lu et al. [50] performed experiments on IN 718 alloy where the specimen with 2 mm by 2 mm island size showed the lowest relative density and most numbers of poles comparing to other specimens with larger island size, which could indicate the lack of full melting problem as shown in the model. Their results showed that with island size lager than 5 mm by 5 mm, the relative density of the build got more independent to the island size. In the same experiment, a crack is also presented in the specimen with 2 mm by 2 mm island size, which was explained by the high frequency of heating and cooling. This high frequency resulted in stress accumulated at the edge of the island. The proposed model quantitatively showed that larger island size leads to less frequent heating and cooling cycle by comparing the temperature peak time for different tracks, which can serve as the foundation for further stress and deflection modal. For the relative density and cracking problem, one solution to this problem is to have an outer contour scan around the island [41], which is not discussed in this paper but worth studying in the future.

**Figure 14:**
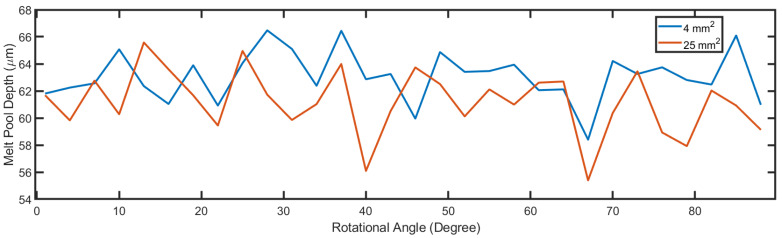
Predicted melt pool depth with different rotational angle between layers applied.

As for the melt pool depth, Figure 14 shows the predicted depth under different rotation angles between layers with same process parameters as in Figure 11. The maximum predicted depth is 66.4 μm at rotation angle of 28 degree and the minimum predicted depth is 58.4 μm at rotation angle of 67 degree. The average predicted depth is 62.7 μm. Similar to the scan direction, the rotational angle between layers does not have essential influence on the melt pool depth but changes the temperature profile significantly. The melt pool depth difference brought by rotation angle between layers reflected the temperature history difference as mentioned before. Different rotational angles bring inconsistent track length across one layer, which leads to different melting time and temperature profile. This further affects the thermal properties on point on the built and creates different melt pool. It is worth noticing that this process considers both the angle between layers and the island dimension. In precious analysis, a uniform 4 ${\mathrm{mm}}^{2}$ island is used for calculation. A larger island with 25 ${\mathrm{mm}}^{2}$ area is also simulated, and the corresponding melt pool depth is also plotted in Figure 14. With larger island size and the same process parameters, the melt pool depth still shows independence respect to the rotation angle between layers. If a smaller island is proposed, a curtain track could be too short to form a stable melt pool at curtain angle, thus break the randomness. Song et al. [34] performed both FEM simulation and experiments on Ti-6Al-4V with different rotational angle between layers. Their results also showed little difference on the melt pool geometries but significant changes on the temperature profile, which is consistent with the result of current model. The size of the melt pool is mainly affected by the applied laser power and scan speed. Even though the previous temperature profile plays a part of the melt pool geometry formation, it is not the essential element. It is worth mentioning that, unlike in most of the FEM models [12,13,14,34], the current model does not consider the boundary condition. The part is still considered as a semi-infinite media, where the heat loss at the boundary is neglected. The proposed model is in better use when the island is at the non-boundary area of the part. However, it can still show the difference between the previous temperature profile and current temperature profile, which indicates the thermal gradient of the point of interest. It is very important for the stress accumulation. The prediction of the temperature profile is important for modelling the residual stress and further deflection [23,24,25,26].

### 4.4. The Effect of Hatch Space on the Temperature History

**Figure 15:**
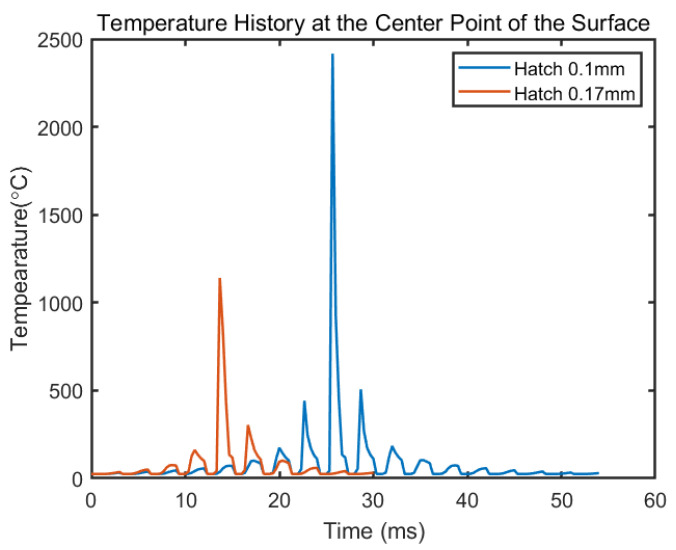
Temperature history of the center point on the fifth layer on a 4 mm^2^ square island under a different hatch space with the same process parameters as in Figure 11.

Figure 15 shows the temperature history of the center point on the surface under different hatching space with other process parameters same as in Figure 11. The value of hatching space changes the number of tracks taken on the same layer, which is represented by the number of peaks in Figure 15. It also elongates the time required for finishing a layer. With a hatch space of 0.17 mm, it takes 30 ms to finish a layer of the island. If the hatch space reduces to 0.1mm, the finishing time becomes longer to 54 ms. The reduction of hatching space significantly increases the number of heating and cooling cycles a point of interest goes through. With smaller hatching space, more heat is accumulated in the part and the laser goes closer to the center point as it approaches, which leads to higher maximum temperature in the temperature history. As a result, smaller hatching space leads to deeper melt pool depth as well as more overlap between melt pools. This is consistent with the trend gained in other SLM research [51]. The overlap of tracks is important to avoid porosity of the build. An inappropriate chosen hatch space may lead to cracks between tracks and incomplete melting [52]. A possible defect of current model the inconsideration of the laser spot diameter. In the current mode, the laser spot is considered as a single point, which will affect the calculated melt pool geometry, especially when the laser spot is significantly large comparing to the melt pool. In that case, the actual melt pool will be wider than the predicted and thus affect the overlap between tracks.

## 5. Conclusions

This paper proposed a predictive analytical temperature model for SLM process that considers the scan strategies, such as the uni- and bi-directional scan strategy on the same layer. It also included the rotational angle between layers as one variable for temperature prediction, which has not been studied in analytical modelling. This leads the analytical model closer to the practical scan situation in industries. The proposed model adopts the transient moving heat source solution which is based on a moving coordinate. A general stationary coordinate is introduced to account for the part dimension and further investigation of the scan direction difference between layers. The proposed model is validated by comparing with two sets of prior experimental data. Sensitivity analysis for the model is also performed and here are some conclusions can be drawn:

- The increase of applied laser power leads to deeper melt pool while the rotational angle between layers is applied. The decrease of scan speed brings similar effect. It is consistent with other researchers [13,14] done without rotation angle. The application of the rotation angle does not affect general heat absorption trend.
- The impact of uni- and bi-directional scan strategy shows more on the temperature profiles across the part, but it is not significant on the melt pool depth. The calculated melt pool depth difference is less than 1%.
- The rotational angles between layers affect both the track number taken on a layer and the single-track length. This leads to different temperature history on a specific point on the part. It does not vary the melt depth in a large scale but may affect the residual stress and further deflection of the part.
- For a 4 ${\mathrm{mm}}^{2}$ island, the short track length due to rotational angle between layers 67 degrees may result in incomplete melt at corner and edge of the island. This can be solved by an extra contour melting [41].
- Smaller hatching space enlarges the time needed to finish a layer scan. It also creates more heat accumulation on the part, which leads to a higher maximum temperature for the center point of the island.

For future work, the thermal history across the build is a foundation for further microstructure modelling and stress modelling. The proposed model could be used as a preliminary step for further analytical modelling work for porosity as well as deflection and other aspects of AM production.

## Figures and Tables

**Table 1:** Material Properties of AlSi10Mg [36,37,38].

Property	Value
Density ρ ($\mathrm{kg}/m^{3}$)	2670
Melting Temperature $T_{\mathrm{melt}}\;$ (℃)	580
Latent Heat of Fusion ${\;L}_{f}$ (J/kg)	423,000
Thermal Conductivity k (W/mC)	$k\left(T\right)=\{\begin{array}{c} 0.0788*T+140.36,\;\;T<488\\ -0.9049*T+619.97,\;488\;\ll \;T\;\ll \;609\\ 69,\;\;T>609 \end{array}$
Specific Heat ${\;C}_{p}$ (J/kgC)	$C_{p}\left(T\right)=\{\begin{array}{c} 0.7822*T+789.79,\;\;T<580\\ -3.5866*T+3323.69,\;\;580\;\leq \;T\;\ll \;620\\ 1100,\;\;T>620 \end{array}$

**Table 2:** Comparison of the predicted melt pool depth to experimental measured data [41,42].

	**P** **(W)**	**V** **(mm/s)**	**h** **(mm)**	**Rotate 67 Degrees**		**Rotate 0 Degrees**
				Measured Depth (μm)	Predicted Depth (μm)	Predicted Depth (μm)
Sample 3	195	700	0.17	59.2	62.2	70.3
Sample 4	195	800	0.17	60.3	58.4	65.9

## Data Availability

The data presented in this study are available on request from the corresponding author.

## Nomenclatures

$C_{p}$	Specific heat
${\mathrm{Cp}}_{0}$	Specific heat at initial time
${\mathrm{Cp}}_{m}$	$\mathrm{Specific}\;\mathrm{heat}\;\mathrm{at}\;m^{\mathrm{th}}$ laser step
E	Enthalpy
k	Thermal conductivity
$k_{0}$	Thermal conductivity at initial time
$k_{m}$	$\mathrm{Thermal}\;\mathrm{conductivity}\;\mathrm{at}\;m^{\mathrm{th}}$ laser step
$L_{f}$	Latent heat of fusion
n	Layer number
P	Laser power
Q	Volumetric heat source
s	Distance between the heat source to the point of interest
𝑇	Temperature
$T_{\mathrm{room}}$	Room temperature
$T^{0}$	Initial temperature
$T^{m}$	$\mathrm{Temperature}\;\mathrm{at}\;m^{\mathrm{th}}$ laser step
t	Time
$t_{0}$	Initial time
Δt	Time difference between two assumed laser steps
u	Internal energy
V	Laser velocity
v	Heat source velocity
$X_{G}Y_{G}Z_{G}$	General coordinate
$X_{L}Y_{L}Z_{L}$	Laser moving coordinate
α	Laser rotation angle between layers
κ	Thermal diffusivity
η	Laser absorption rate
φ	Laser rotation angle between first layer and current layer
ρ	Material density
ξ	Integration variable
